# Draft Genome Sequence of Fructophilic *Lactobacillus florum*

**DOI:** 10.1128/genomeA.00025-12

**Published:** 2013-01-24

**Authors:** Eun Bae Kim, Charlotte A. Tyler, Lauren M. Kopit, Maria L. Marco

**Affiliations:** Department of Food Science & Technology, University of California, Davis, California, USA

## Abstract

Herein we report the first genome sequence for *Lactobacillus florum*. *L. florum* 2F was isolated from Valencia orange leaves and is fructophilic, like other strains of this species. The draft genome of *L. florum* 2F contains 1,261,842 bp with a *G+C* content of 41.5% in 46 contigs (≥500 bp).

## GENOME ANNOUNCEMENT

In 2010, the species *Lactobacillus florum* was first described for novel bacterial isolates originating from peony and bietou flowers in South Africa (1, 2). Several other *L. florum* strains have since been isolated from grapes and wine (3). *L. florum* is most closely related to *Lactobacillus lindneri* and *Lactobacillus sanfranciscensis*, but it differs from these species because *L. florum* is fructophilic and exhibits a preference for d-fructose but not d-glucose, a primary carbon source for most lactobacilli (2).

Strain 2F was isolated along with other lactic acid bacteria (LAB) from the leaves and (un)ripe fruits of (semi)tropical plants in California, including cactus, rose apple, tangelo, and Valencia orange. Most of the 79 *L. florum* isolates recovered exhibited a growth preference for fructose rather than glucose on MRS agar. *L. florum* 2F was among the isolates from Valencia orange leaves. This strain was selected for genome sequencing because it exhibited the most common random amplified polymorphic DNA (RAPD)-PCR genotype pattern shared among the *L. florum* isolates recovered from plants.

To prepare genomic DNA, one colony of *L. florum* 2F was inoculated into 5 ml of fructose-containing MRS broth and incubated at 30°C for 12 h. *L. florum* cells were harvested by centrifugation at 5,000 × *g* for 10 min and then washed twice with phosphate-buffered saline (PBS). Genomic DNA was then purified using the DNeasy blood and tissue kit (Qiagen, Valencia, CA). A 500-bp insert library was constructed for paired-end 100-bp sequencing (2 × 100 bp). The library was sequenced using Illumina HiSeq 2000 at the UC Davis Genome Center (Davis, CA). The sequences were quality filtered, resulting in 1,056 Mbp representing an 837.2-fold coverage of the genome. The reads were then assembled by Velvet 1.2.07 (4) with a k-mer size of 31 bp to generate 46 contigs (≥500 bp; total length, 1,261,842 bp; N50 length, 59,916 bp; average length, 27,431 bp; *G+C* content, 41.5%). A total of 1,190 coding sequences (CDS) and 18 tRNAs were predicted by Rapid Annotation Using Subsystem Technology (RAST) (5). The strain lacks known genetic loci coding for antibiotic resistance based on comparisons to 22,190 genes in the Antibiotic Resistance Genes Database (6). We detected four clustered regularly interspaced short palindromic repeats (CRISPR)-associated genes (*cas1*, *cas2*, and one SAG0894 and SAG0897 family gene), which indicates that the *L. florum* has an acquired immunity against foreign genetic elements (for example, bacteriophages) as found for other LAB species (7). Two genes encoding fructokinase and glucose-6-phosphate isomerase, essential for fructose metabolism, are present and highly conserved (76% and 74% nucleotide identities, respectively) when compared to *L. sanfranciscensis* (8). Genes for utilizing plant cell wall degradation products such as arabinose and xylose are lacking, unlike those of other plant-associated LAB (e.g., *Lactococcus lactis* KF147) (1, 9). These genetic comparisons are useful for understanding the adaptations of LAB to plant-associated environments.

### Nucleotide sequence accession numbers.

This whole-genome shotgun project has been deposited at DDBJ/EMBL/GenBank under the accession number ALXF00000000. The version described in this paper is the first version, ALXF01000000.
